# Networks and long-range mobility in cities: A study of more than one billion taxi trips in New York City

**DOI:** 10.1038/s41598-020-60875-w

**Published:** 2020-03-04

**Authors:** A. P. Riascos, José L. Mateos

**Affiliations:** 1 Instituto de Física, Universidad Nacional Autónoma de México, Apartado Postal 20-364, 01000 Ciudad de México, Mexico; 2 Centro de Ciencias de la Complejidad, Universidad Nacional Autónoma de México, Apartado Postal 04510, Ciudad de México, Mexico

**Keywords:** Applied physics, Complex networks

## Abstract

We analyze the massive data set of more than one billion taxi trips in New York City, from January 2009 to December 2015. With these records of seven years, we generate an origin-destination matrix that has information of a vast number of trips. The mobility and flow of taxis can be described as a directed weighted network that connects different zones of high demand for taxis. This network has in and out degrees that follow a stretched exponential and a power law with an exponential cutoff distributions, respectively. Using the origin-destination matrix, we obtain a rank, called "*OD rank*”, analogous to the page rank of Google, that gives the more relevant places in New York City in terms of taxi trips. We introduced a model that captures the local and global dynamics that agrees with the data. Considering the taxi trips as a proxy of human mobility in cities, it might be possible that the long-range mobility found for New York City would be a general feature in other large cities around the world.

## Introduction

The study and understanding of human mobility in cities is an important and challenging problem since more than half of the world population lives in urban areas^1^. Nowadays human mobility can be explored in detail thanks to the digital traces people leave on mobile/digital platforms^2,3^. Identifying global emerging patterns for human mobility is important in topics like urban planning, transport systems, the influence of the spatial distribution of a city in the mobility^4–7^, and the encounter or contact networks that emerge^8^. In addition to all these aspects lying in the field of complexity and cities, we have the science of networks with well-established tools and methods to describe complex systems^9–11^. In many cases, networks provide an important framework to study transportation modes and their interactions^12,13^.

Several studies have revealed that human mobility follows a long-range dynamics, akin to Lévy walks, as has been shown before as a common strategy in many animal species and humans^3,14^. In the context of networks, Lévy flights were introduced in^15^ revealing that long-range displacements increase the capacity to reach efficiently to any site of the network by inducing the small-world property through the dynamics. This process has been explored in different cases as diverse as fractional diffusive transport^16–19^, the dynamics on multiplex networks^20^, human mobility^8,21^, semi-supervised learning^22^, among others^19,23–27^.

In this research, we analyze the spatial activity of taxis as a proxy for human mobility in urban areas. From publicly available datasets, we generate an origin-destination (OD) matrix for trips during a period of seven years from January 2009 to December 2015. We identify zones with a high demand of this service and in this way, the movement of taxis can be described as a directed weighted spatial network with nodes representing high demand zones and links defined by the number of trips between two zones. In addition, we have geographic coordinates for all the nodes and the respective distances between them; as a result, the system can be described as a spatial network^28^. With all this information, available through the analysis of trip records, we study the spatial activity of taxis as a dynamical process in this particular structure. Several authors have explored spatio-temporal patterns in the mobility of taxis in different urban areas^29–31^. The system of taxis in New York City has been studied with different methods; in particular, considering the complete routes followed by the taxis on the street network^32–35^.

**Figure 1:**
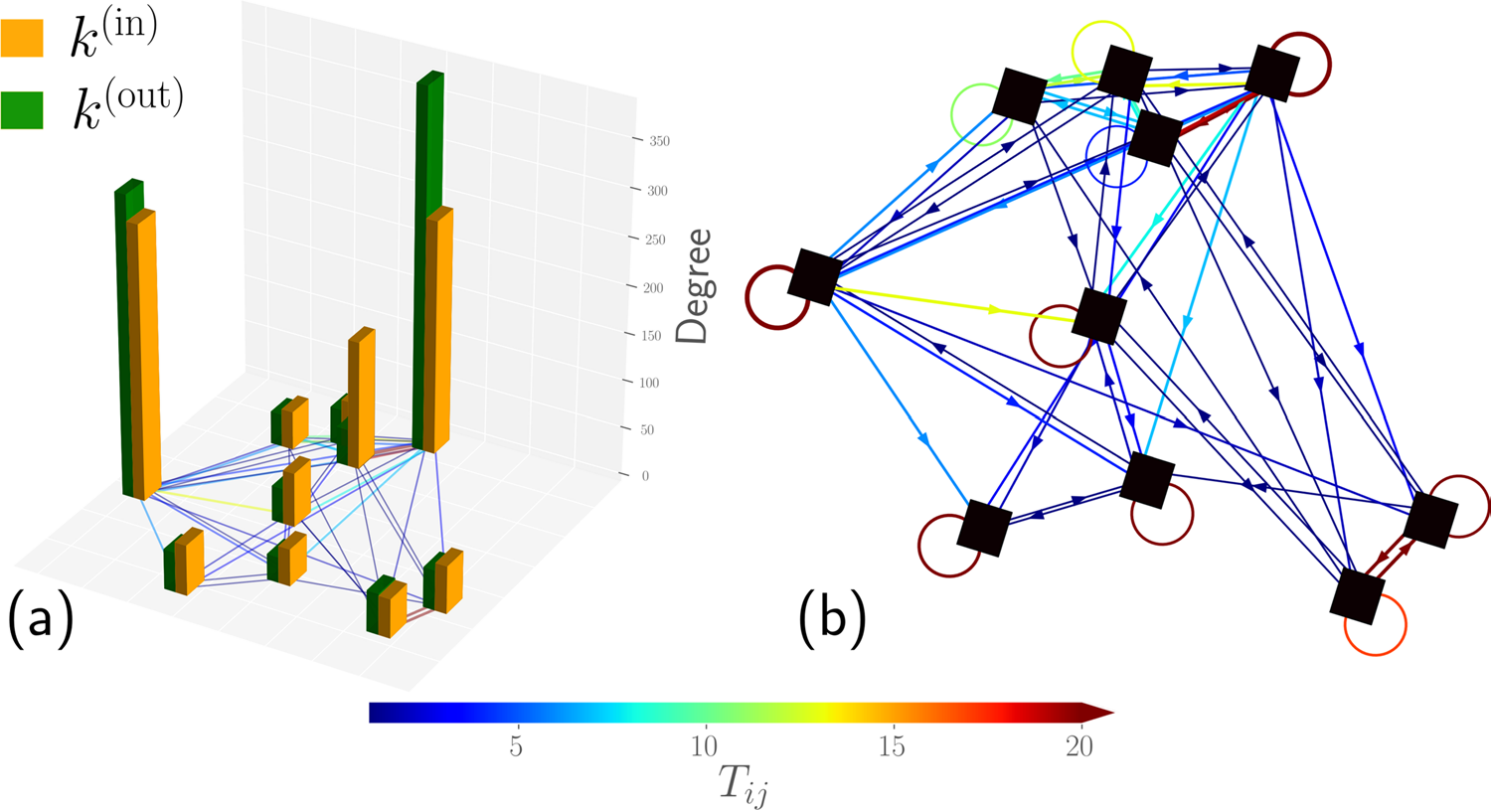
Schematic illustration of mobility as a spatially embedded directed weighted network. We show *N* = 10 square zones in the plane representing particular regions where agents can start or end a trip; we simulate ${\mathscr{T}}=1\ 000$ trips of agents between these locations. (**a**) Bar representation of the total number arrivals *k*^(in)^ and the number of departures *k*^(out)^. (**b**) Diagram of the system expressed as a directed network, we represent with colors the number of trips *T*_*ij*_ between sites *i* and *j*. In the study of human mobility, this information is expressed as an origin-destination matrix *N* × *N* with elements *T*_*ij*_. In particular, the directions of the links are depicted by arrows and self-loops represent the number of trips with the same origin and destination.

To clarify the connection between mobility and networks, let us illustrate some ideas in connection with the relation between directed weighted networks and human mobility. In Fig. 1 we depict a schematic illustration of agents moving between *N* = 10 specific regions denoted as squares in a two-dimensional plane. In this reduced example, we have ${\mathscr{T}}=1000$ trips and the values *T*_*ij*_ (for *i*, *j* = 1, 2, …*N*) denote the number of trips between two regions. In Fig. 1(a) we represent with bars the values obtained for the number of trips that arrive or depart each zone; in addition, colored lines denote the number of trips *T*_*ij*_. In Fig. 1(b) we represent the complete structure described by the origin-destination matrix as a directed weighted network: the links have directions represented by an arrow and, with different colors in the lines, we depict the respective number of trips. Furthermore, we show with self-loops (i.e., a line connecting a zone with itself) the number of trips that start and end in the same zone, determined by the diagonal elements in the origin-destination matrix. In addition, in the study of mobility, the resulting structure is a spatial network and all the positions of the nodes are important, for instance, to determine the distance between two zones. This example shows the vast amount of information that is captured in the origin-destination matrix and its direct relation to a network, allowing us to use the full potential of network science to study mobility.

The paper is organized as follows. In the first part, we identify high demand zones and generate origin-destination matrices describing the global activity of taxi’s flow. Then, we calculate the transition probabilities between high demand zones. We introduce a rank, called "*OD rank*”, analogous to the page rank of Google. We also implement a model that describes the spatial activity of taxis and verify the predictions of this model with the real data through Monte Carlo simulations. Our findings reveal a well defined mathematical structure for the spatial mobility in urban areas with a dynamics that combines local displacements with a particular type of long-range movements. The methods introduced are general and can be used as a framework for the study of different transportation systems in cities.

## Results

### Activity between zones with high demand

**Figure 2:**
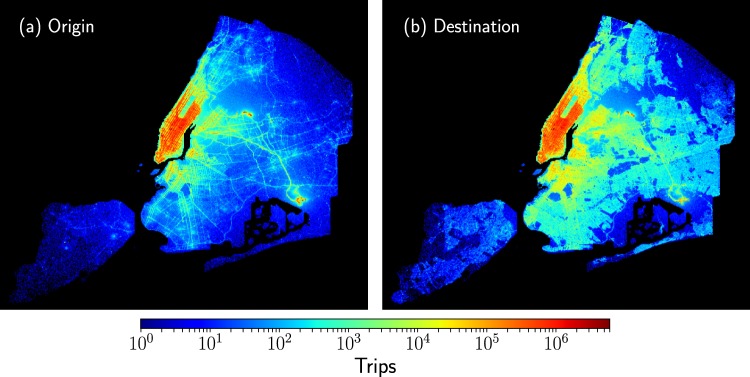
Origins and destinations of taxi trips in New York City. In this analysis, we divide the city in 100 m × 100 m square regions and, for each region, we count the number of taxi trips considering the registers of longitude and latitude of the initial and final locations of each trip. The results are presented in (**a**) for the origins and (**b**) for the destinations of taxis. The colorbar indicates the number of trips in each zone; regions outside the boundaries of New York City are presented in black. We analyze ${\mathscr{T}}=1\ 148\ 050\ 837$ trips from taxi trip records between January 2009 to December 2015. In this representation of the data, using only the information of origins and destinations of taxis, we can see in detail the spatial complexity of New York City and how the street network emerges from the large number of trips analyzed.

We explore taxi trip records taking into account the administrative boundaries including the five boroughs of New York City^36^. As a result, for the seven years studied, we have ${\mathscr{T}}=1\ 148\ 052\ 357$ taxi trips (see the Methods section for a detailed description of the datasets explored). In the following, we study this volume of data by using a grid with 500 × 500 square zones with dimensions 100 m × 100 m. Once this grid is defined, we examine the zones contained in the administrative boundaries of New York City. In Fig. 2, we present a map generated with the information of origin and destinations reported in the datasets. For each square zone defined before, we count the number of trips according to the registers of longitude and latitude of the initial and final locations of each trip for taxi registers from 2009 to 2015. The results depicted in Fig. 2 give us a first insight into the global activity of taxis. We can identify a high demand of this service in Manhattan, also the high activity in the John F. Kennedy (JFK) International Airport and how by exploring the origins of the trips we can detect some features of the street network in New York City. On the other hand, we can see in Fig. 2(b) that the destinations are less localized in specific zones observing that in the Bronx, Brooklyn and Queens boroughs the number of taxis arrivals is more uniform in comparison with the origins in Fig. 2(a). This particular feature reveals how taxi transportation manages to permeate almost all the regions of the city.

In Fig. 2, we can identify zones in New York City with low demand for taxis or where only a reduced number of taxis arrives. Even considering the counts in seven years of activity, we can identify zones with dimensions 100 m × 100 m for which less than 10^3^ taxi trips arrive or depart. This is a small number in comparison with the values of zones with a high demand for which we observe more than a million arrivals or departures. Much of these zones are located in Manhattan but also other zones of the city. In what follows, we study the flow of taxis between zones with high demand and we will describe the global spatial dynamics as a directed weighted spatial network. All the zones in our study are defined by a square with dimensions 100 m × 100 m and, for each year, we classify a region as a high activity zone if, in this specific part of the city, the number of arrivals and departures are at least 10^3^. In this way, the minimum number of arrivals at a high activity zone is at least 10^3^ trips, and the same rule applies to the number of taxis leaving this region. This limit is reasonable due to the high quantity of trip records explored per year in the complete database. In addition, by using this rule we reduce possible errors produced by the inaccuracy in the origin and destination coordinates. By applying the criteria described before to the taxi trips in 2015, the number of high demand zones for this year is 4 353 and the total number of trips between these zones is ${\mathscr{T}}=128\ 984\ 657$ that represents a 90.22% of the original database described in the Methods section. We found similar values for the trips from 2009 to 2014. The results for the number of high demand zones *N* and the total number of trips ${\mathscr{T}}$ are presented in Table 1.

**Table 1:** Analysis of the spatial activity of taxi trips in New York City considering zones with a high demand for this service. By using the rule that at least ${\mathscr{M}}=1\ 000$ trips depart and arrive from a zone in a year, we obtain the number *N* of high demand zones. In addition, we present the total number of trips ${\mathscr{T}}$ between zones and the fraction of the original dataset that each number of trips represents. For the trips analyzed, we show the fraction of local trips with geographical distances *d* in the interval 0 ≤ *d* ≤ 1.8 Km and the fraction of long-range movements with *d* > 1.8 Km.

Year	Fraction original database (%)	*N*	Displacements ${\mathscr{T}}$	0 ≤ *d* ≤ 1.8 Km (%)	*d* > 1.8 Km (%)
2009	91.76	4 456	153 389 115	44.69	55.31
2010	91.71	4 465	150 327 196	43.87	56.13
2011	91.70	4 558	156 962 079	42.92	57.08
2012	91.70	4 642	158 714 293	42.44	57.56
2013	91.65	4 645	154 833 137	42.69	57.31
2014	91.08	4 612	146 484 526	43.39	56.61
2015	90.22	4 353	128 984 657	43.21	56.79

Now, we define origin-destination matrices describing the flow of taxis between high-demand zones. In this way, the global dynamics can be explored and treated as a directed weighted network; in particular, a spatial network for which the nodes represent zones of high demand and the links with weights can represent several quantities like the flow of vehicles, the geographical distance between nodes, among other values^28^.

For each year, we calculate an origin-destination matrix for which the elements *T*_*ij*_ represent the number of taxi-trips from zone *i* to zone *j*, where *i*, *j* = 1, 2, …, *N* denote the square zones of high demand with dimensions 100 m × 100 m. In addition to the elements of the origin-destination matrix, it is important the in-degree defined as

1 $${k}_{i}^{({\rm{in}})}=\mathop{\sum }\limits_{\ell =1}^{N}{T}_{\ell i}$$

that gives the total number of vehicles arriving at the zone *i*. We also have the out-degree determined by the relation

2 $${k}_{i}^{({\rm{out}})}=\mathop{\sum }\limits_{\ell =1}^{N}{T}_{i\ell },$$

that counts the total number of trips originated from zone *i*. On the other hand, to explore the spatial activity is important to have information about the geographical distances between zones. This information is included in a *N* × *N* distance matrix **D** with elements *d*_*ij*_ with the geographical distance between *i* and *j*. In addition, the degrees in Eqs. 1–2 satisfy:

3 $$\mathop{\sum }\limits_{i=1}^{N}{k}_{i}^{({\rm{o}}{\rm{u}}{\rm{t}})}=\mathop{\sum }\limits_{i=1}^{N}{k}_{i}^{({\rm{i}}{\rm{n}})}=\mathop{\sum }\limits_{\ell =1}^{N}\mathop{\sum }\limits_{m=1}^{N}{T}_{\ell m}={\mathscr{T}},$$

where ${\mathscr{T}}$ is the total number of trips considered in the origin-destination matrix.

**Figure 3:**
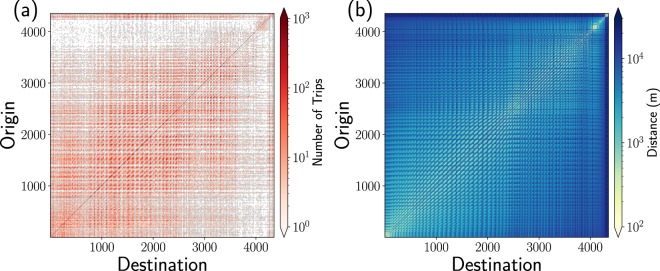
Global activity of taxis between zones of high demand for this service in New York City. We analyze the movement of taxis trips made in 2015 and, from the study of a grid with square zones with 100 m × 100 m, similar to the one presented in Fig. 2, we identify zones of high demand of taxis considering that at least 1 000 trips have departed or arrived from a zone. We found with this criterion *N* = 4 353 high demand zones. In (**a**) we present the origin-destination matrix for taxi trips moving between zones, the respective colorbar codifies the trip counts. In (**b**) we present the geographical distance between origin and destination zones; the values of the distance are represented in the colorbar.

In Fig. 3, we show the origin-destination matrix and the respective matrix of distances **D** obtained from taxi trips in 2015. The resulting matrices incorporate the flow of vehicles between *N* = 4353 high demand zones.

**Figure 4:**
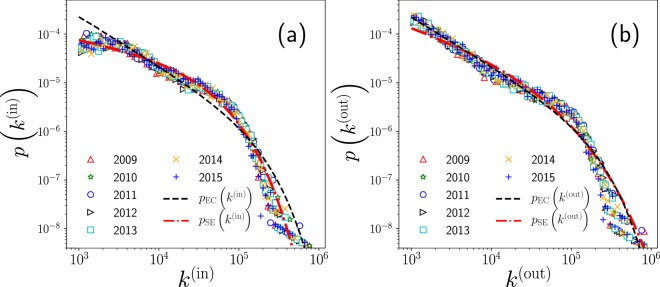
Statistical analysis of the number of taxi trips that depart and arrive in high demand zones in New York City. We present the probability density for the values of the degrees: (**a**) ${k}_{i}^{({\rm{in}})}$ and (**b**) ${k}_{i}^{({\rm{out}})}$ defined in Eqs. 1–2 for *i* = 1, 2, …, *N*, where *N* is the number of high activity zones presented in Table 1 for each of the years explored. The results were obtained with normalized counts using logarithmically spaced bins. In both cases, we show with different curves, the power law with an exponential cutoff *p*_EC_(*k*) in Eq. 4 and the stretched exponential fit *p*_SE_(*k*) in Eq. 5.

Let us now analyze the statistical properties of the directed weighted network associated with mobility in New York City. In order to do so, we show in Fig. 4 two probability distributions: one associated to the in-degree of the network ${k}_{i}^{({\rm{in}})}$ (Fig. 4(a)) and the other one associated with the out-degree of the network ${k}_{i}^{({\rm{out}})}$ (Fig. 4(b)). We explore all the in and out-degrees, for seven years, from 2009 to 2015, in the interval 10^3^ ≤ *k* ≤ 10^6^. With the aim of finding the best fit of the aggregated data of mobility for these distributions, we used the tools and procedures described by Clauset *et al*. (2009) as given in ref. ^37^, that are implemented in the *powerlaw* package library described in references^38–40^. In order to decide the best fit and perform a proper statistical analysis, we explore several candidates for the distribution models: power law, power law with an exponential cutoff, exponential, stretched exponential and log-normal.

For the statistical distributions considered, we calculate the Kolmogorov-Smirnov (KS) distance between them in a pairwise fashion. This KS distance gives us a first indicator (goodness of fit) of the proximity of the data and the proposed distribution model. Then, we compare the different distributions via a likelihood ratio test by calculating the log-likelihood function of each one of the selected distributions. The sign of this ratio gives us a criterion to discriminate between distributions (see reference^37^). After this model selection, the best two fits were the power law with an exponential cutoff (EC), with a probability density^37^:

4 $${p}_{{\rm{EC}}}(k)=\frac{{\lambda }^{1-\gamma }}{\Gamma (1-\gamma ,\lambda {k}_{\min })}{k}^{-\gamma }{e}^{-\lambda k}$$

and the stretched exponential (SE)^37^:

5 $${p}_{{\rm{SE}}}(k)=\beta \lambda {k}^{\beta -1}{e}^{-\lambda ({k}^{\beta }-{k}_{\min }^{\beta })}$$

where *k* represents the degree, ${k}_{\min }$ is the minimum value considered in the fit and Γ(*x*, *y*) denotes the incomplete gamma function. Notice that both distributions have two parameters, that we will distinguish with a superindex EC for the power law with an exponential cutoff, and with a superindex SE for the stretched exponential; however, we will not indicate these superindexes in Eqs. 4–5 to simplify the notation. We use *λ*^EC^ and *γ*^EC^ for the power law with an exponential cutoff and *λ*^SE^ and *β*^SE^ for the stretched exponential.

For the in-degrees in Fig. 4(a), the best fit is the stretched exponential with parameters ${\beta }_{{\rm{in}}}^{{\rm{SE}}}=0.708$ and ${\lambda }_{{\rm{in}}}^{{\rm{SE}}}=4.138\times 1{0}^{-5}$; in a similar way, for the power law with exponential cutoff ${\gamma }_{{\rm{in}}}^{{\rm{EC}}}=1.00000000041$ and ${\lambda }_{{\rm{in}}}^{{\rm{EC}}}=6.730\times 1{0}^{-6}$. On the other hand, the same analysis for the out-degrees in Fig. 4(b) concludes that the best fit is the power law with an exponential cutoff with parameters ${\gamma }_{{\rm{out}}}^{{\rm{EC}}}=1.0000000025$ and ${\lambda }_{{\rm{out}}}^{{\rm{EC}}}=6.086\times 1{0}^{-6}$; in addition, for the stretched exponential ${\beta }_{{\rm{out}}}^{{\rm{SE}}}=0.495$ and ${\lambda }_{{\rm{out}}}^{{\rm{SE}}}=6.834\times 1{0}^{-5}$.

It is surprising that both exponents ${\gamma }_{{\rm{in}}}^{{\rm{EC}}}$ and ${\gamma }_{{\rm{out}}}^{{\rm{EC}}}$ are extremely close to the value one. Thus, both distributions are well described by the power law *p*(*k*) ∝ *k*^−1^ in some range of in and out degrees.

### Transition probabilities

All the information in the origin-destination matrix and in the degrees ${k}_{i}^{({\rm{in}})}$ and ${k}_{i}^{({\rm{out}})}$ allow us to analyze and understand the spatial activity of taxis as a dynamical process in a spatial directed weighted network. In this way, we can describe statistically the global dynamics of taxis in terms of transition probabilities between high demand zones of this service.

The transition probability ${w}_{i\to j}^{({\rm{out}})}$ between zones *i* and *j* is defined in terms of the origin-destination matrix as:

6 $${w}_{i\to j}^{({\rm{out}})}=\frac{{T}_{ij}}{{k}_{i}^{({\rm{out}})}}.$$

With this definition, the transition probabilities satisfy the normalization condition:

7 $$\mathop{\sum }\limits_{\ell =1}^{N}{w}_{i\to \ell }^{({\rm{out}})}=1.$$

With the transition probabilities ${w}_{i\to j}^{({\rm{out}})}$, we can explore the relationship between the information in the origin-destination matrix and the matrix of distances; these matrices were presented in Fig. 3. Now, to study this connection, we calculate ${w}_{i\to j}^{({\rm{out}})}$ by using the definition in Eq. 6; for each value, we have the corresponding geographical distance *d*_*ij*_ between *i* and *j* as an entry in the distance matrix **D**.

**Figure 5:**
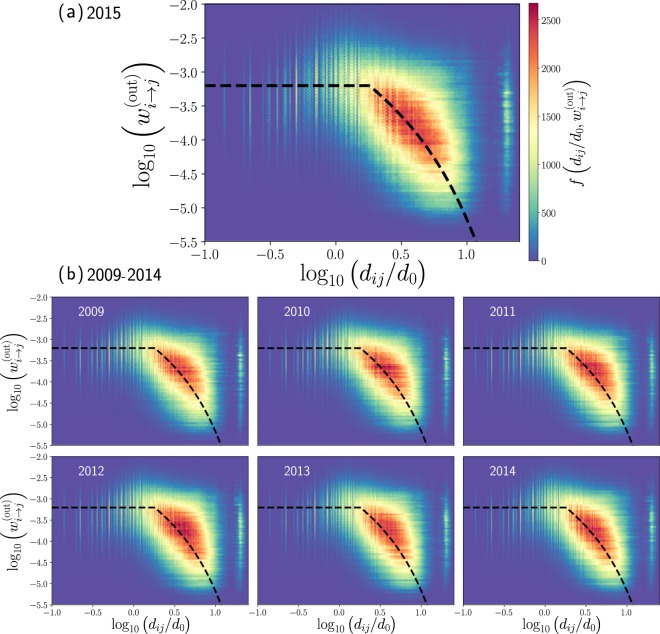
Transition probabilities between zones of taxi trips in New York City. In (**a**) we present the results obtained for the year 2015 with origin-destination matrix and the respective distances presented in Fig. 3. In (**b**) we depict our findings for each year from 2009 to 2014. In all these cases, we analyze the non-null transition probabilities ${w}_{i\to j}^{({\rm{out}})}$ and the geographical distance *d*_*ij*_ between zones *i* and *j*. We show hexagonally binned two-dimensional histograms for the logarithm of ${w}_{i\to j}^{({\rm{out}})}$ and the logarithm of *d*_*ij*_/*d*_0_ where *d*_0_ = 1 Km is a reference distance. The values codified in the colorbar represent the frequencies denoted as $f({d}_{ij}/{d}_{0},{w}_{i\to j}^{({\rm{out}})})$ of the pairs $\left({{\rm{\log }}}_{10}\left(\frac{{d}_{ij}}{{d}_{0}}\right),{{\rm{\log }}}_{10}{w}_{i\to j}^{({\rm{out}})}\right)$ found in each hexagonal bin. Dashed lines are used as a guide and represent the behavior ${w}_{i\to j}^{({\rm{out}})}$ constant, for *d* ≤ 1.8 Km, and ${w}_{i\to j}^{({\rm{out}})}\propto {d}_{ij}^{-1}{e}^{-\beta ({d}_{ij}-R)}$ with *β* = 0.15 Km^−1^ for *d* > 1.8 Km.

In Fig. 5, we depict the logarithm of the transition probability ${w}_{i\to j}^{({\rm{out}})}$ as a function of the logarithm of the relation *d*_*ij*_/*d*_0_ where *d*_0_ = 1 Km is a reference length. In Fig. 5(a), we consider all the non-null transition probabilities ${w}_{i\to j}^{({\rm{out}})}$ and distances *d*_*ij*_, for the annual data records of the taxi’s activity in 2015; we obtain a distribution of points $\left({{\rm{\log }}}_{10}\left(\frac{{d}_{ij}}{{d}_{0}}\right),{{\rm{\log }}}_{10}{w}_{i\to j}^{({\rm{out}})}\right)$ for all the zones with high demand (*i*, *j* = 1, 2, …, *N*). We show the results as a two-dimensional histogram that quantifies the frequencies of these values in hexagonal bin counts.

**Figure 6:**
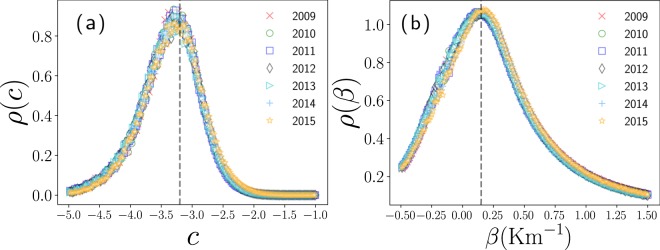
Statistical analysis of the parameters *c* and *β*. We present the probability density *ρ* of the numerical values *c* and *β* found for each pair $\left({{\rm{\log }}}_{10}\left(\frac{{d}_{ij}}{{d}_{0}}\right),{{\rm{\log }}}_{10}{w}_{i\to j}^{({\rm{out}})}\right)$ in the interval 0.1 Km ≤ *d*_*ij*_ ≤ 11 Km for the years 2009, 2010, …, 2015. (**a**) Values $c={{\rm{\log }}}_{10}{w}_{i\to j}^{({\rm{out}})}$ for *d*_*ij*_ ≤ *R* = 1.8 Km, (**b**) values *β* obtained from Eq. 8 for *d*_*ij*_ > *R*. Vertical dashed lines represent the values *c* = −3.2 and *β* = 0.15 Km^−1^.

Our findings in Fig. 5 reveal that the transition probabilities of taxis are approximately constant ${w}_{i\to j}^{({\rm{out}})}=1{0}^{c}$ for distances less than a characteristic value *R* = 1.8 Km. In contrast, for distances greater than *R*, the transition probabilities are well described by a power law with an exponential cutoff relation:

8 $${w}_{i\to j}^{({\rm{out}})}=a\frac{R}{{d}_{ij}}{e}^{-\beta ({d}_{ij}-R)}\qquad {\rm{for}}\qquad {d}_{ij} > R,$$

where continuity for *d*_*ij*_ = *R* requires *a* = 10^*c*^. Now, to find the best fit, we analyze the pairs  $\left({{\rm{\log }}}_{10}\left(\frac{{d}_{ij}}{{d}_{0}}\right),{{\rm{\log }}}_{10}{w}_{i\to j}^{({\rm{out}})}\right)$ presented in Fig. 5 for values 0.1 Km ≤ *d*_*ij*_ ≤ 11 Km. We divide the data considering pairs in the region *d*_*ij*_ ≤ *R* and *d*_*ij*_ > *R* with *R* = 1.8 Km. In Fig. 6(a) we show the statistical analysis of the values $c={{\rm{\log }}}_{10}{w}_{i\to j}^{({\rm{out}})}$ found for *d*_*ij*_ ≤ *R*, we see that the values *c* are distributed with a pronounced peak around *c* = −3.2, we use this value to describe the probabilities of transition. In a similar way, once defined *c*, we calculate *β* in Eq. 8 for *d*_*ij*_ > *R*. In Fig. 6(b), we analyze the probability density of the values *β* and we identify a peak around *β* = 0.15 Km^−1^.

The piecewise approximations described by the values *R* = 1.8 Km, *c* = −3.2 and *β* = 0.15 Km^−1^ are represented with dashed lines in Fig. 5. A similar behavior has been detected in the analysis of the transportation network of stations in bicycle sharing systems operating in New York City and Chicago^21^. In these cases, the value *R* ≈ 1 Km defines local displacements and the long-range dynamics is well described by ${w}_{i\to j}^{({\rm{out}})}\propto {d}_{ij}^{-2}$. In this way, in bike-sharing systems *R* is reduced in comparison to our findings for taxi trips; in addition, the long-range spatial activity qualitatively has similar characteristics to those observed in Fig. 5.

### OD rank

The transition matrix **W**^(out)^ with elements ${w}_{i\to j}^{({\rm{out}})}$ defined in Eq. 6 allow us to understand human mobility as a dynamical process in a spatial directed weighted network. Well-known results in stochastic processes apply for the transition matrix **W**^(out)^^10^. In most cases, origin-destination matrices are non-symmetric; as a consequence, it is convenient to analyze the transition matrix **W**^(out)^ establishing an analogy with the Google matrix^41^, with a mathematical structure entirely general that applies to any graph or network in any domain^42^. In the following, we explore how by using this connection, the eigenvalues and eigenvectors of **W**^(out)^ give valuable information to understand the movement of taxis.

The transition matrix **W**^(out)^ has left and right eigenvectors. Left eigenvectors ${\overrightarrow{\Phi }}_{j}$ with elements *ϕ*_*j*_(*i*) satisfy:

9 $${\overrightarrow{\Phi }}_{j}{{\bf{W}}}^{({\rm{out}})}={\lambda }_{j}{\overrightarrow{\Phi }}_{j}\qquad {\rm{for}}\qquad j=1,2,\ldots ,N,$$

where ${\{{\lambda }_{j}\}}_{j=1}^{N}$ are the eigenvalues of the transition matrix. Right and left eigenvectors form an orthonormal base and have the same eigenvalues. On the other hand, the stochastic matrix **W**^(out)^ fulfills Eq. 7 and, by definition, the elements of *T*_*ij*_ satisfy *T*_*ij*_ ≥ 0; therefore, **W**^(out)^ belongs to the class of Perron-Frobenius operators with a possibly degenerate unit eigenvalue *λ* = 1 and other eigenvalues obeying ∣*λ*∣ ≤ 1 (see^43^ for details).

**Figure 7:**
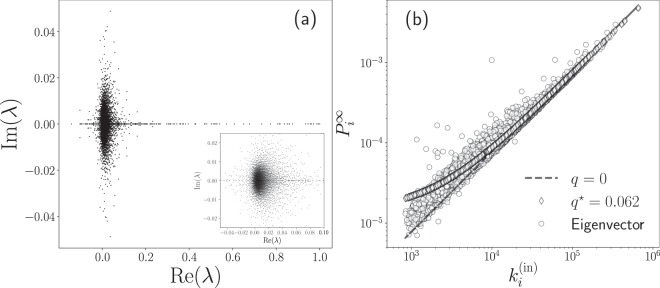
Numerical analysis of the eigenvalues and OD rank of the transition matrix **W**^(out)^. We analyze the transition probability matrix for the taxi’s flow in 2015 with origin-destination matrix presented in Fig. 3 with *N* = 4 353 high demand zones. In (**a**), we show the eigenvalues *λ* of **W**^(out)^ satisfying Eq. 9. In this way, we have 4 353 values represented in the complex plane with dots; in the inset, we depict the results for the eigenvalues in a region close to the origin, where we observe more eigenvalues with a non-null complex part. In (**b**) we plot the components ${P}_{i}^{\infty }$ of the eigenvector ${\overrightarrow{{\bf{P}}}}^{\infty }$ with eigenvalue *λ* = 1; we represent the numerical values of ${P}_{i}^{\infty }$ in terms of the respective degree ${k}_{i}^{({\rm{in}})}$ for *i* = 1, 2, …, *N*. We also show the values ${P}_{i}^{\infty }(q)$ obtained with Eq. 11 for *q* = 0 and the best fit q^*^ = 0.062.

In Fig. 7(a) we plot the eigenvalues of the transition matrix **W**^(out)^ for taxi trips in New York City in 2015. We use the origin-destination matrix in Fig. 3(a) and the definition in Eq. 6. The results were obtained numerically and, due to the asymmetry of the origin-destination matrix, the eigenvalues are complex numbers. In Fig. 7(a) we show the real and imaginary part of each of the eigenvalues *λ*_*i*_ for *i* = 1, 2, …, *N* = 4 353. In this analysis, we found that only one eigenvalue satisfies *λ* = 1, a result that reveals that the directed network associated with the mobility between sites of high demand for taxis is connected. Therefore, the links in the network connect all the zones. This particular result can be interpreted using the terminology of random walks on networks. In this case, the movement of a random walker defined in terms of the transition matrix **W**^(out)^ is capable to visit any node of the network only by moving on the links, independently of the initial configuration. As we mentioned before, the high connectivity observed in the origin-destination matrix is a consequence of considering high demand zones with a criterion that requires a high number of departures and arrivals in each zone avoiding the emergence of isolated parts. However, the approach developed is general and in other cases, similar spectral analysis of the transition matrix could be an important tool to identify disconnected parts in a transportation system.

In addition to the eigenvalues, the respective eigenvectors of the transition matrix provide valuable information about dynamical processes on networks^10,19^. In particular, the left eigenvector associated with the eigenvalue *λ* = 1 defines a ranking vector ${\overrightarrow{{\bf{P}}}}^{\infty }$ with elements ${P}_{i}^{\infty }$ for *i* = 1, 2, …, *N* and satisfies ${\overrightarrow{{\bf{P}}}}^{\infty }{{\bf{W}}}^{({\rm{out}})}={\overrightarrow{{\bf{P}}}}^{\infty }$, therefore:

10 $${\sum }_{\ell =1}^{N}{P}_{\ell }^{\infty }{w}_{\ell \to j}^{({\rm{out}})}={P}_{j}^{\infty }.$$

In the study of random walks on networks, the vector ${\overrightarrow{{\bf{P}}}}^{\infty }$ is the stationary probability distribution. The value ${P}_{i}^{\infty }$ gives the probability of a random walker to reach the node *i* after a large number of steps^19^. In the context of the Google matrix, the vector ${\overrightarrow{{\bf{P}}}}^{\infty }$ determines the importance of a node in a network establishing a PageRank of the Web^43^. In the analysis of mobility with a transition matrix **W**^(out)^, the vector ${\overrightarrow{{\bf{P}}}}^{\infty }$ defines a ranking of the zones used in the definition of the origin-destination matrix. Due to this connection, we call this ranking “*OD rank*”.

In Fig. 7(b), we show the results obtained numerically for the OD rank ${\overrightarrow{{\bf{P}}}}^{\infty }$ associated with the eigenvalue *λ* = 1 of the transition matrix **W**^(out)^ that describes the taxi’s flow in 2015. Our findings in this figure reveal a connection between the OD rank ${P}_{i}^{\infty }$ of a zone *i* and the respective in-degree ${k}_{i}^{({\rm{in}})}$. In a similar way to the findings for the PageRank algorithm for Google, the stationary probability distribution ${\overrightarrow{{\bf{P}}}}^{\infty }$ is a measure of the popularity of nodes that is mostly due to the in-degree dependence; in a mean-field approximation the stationary distribution of the PageRank algorithm is given by^44^:

11 $${P}_{i}^{\infty }(q)=\frac{q}{N}+(1-q)\frac{{k}_{i}^{({\rm{in}})}}{{\mathscr{T}}},$$

where 0 ≤ *q* ≤ 1. Searching the optimal value q^*^ that minimizes the quadratic error $S(q)={\sum }_{\ell =1}^{N}{({P}_{\ell }^{\infty }-{P}_{\ell }^{\infty }(q))}^{2}$, we get for the best fit:

12 $${q}^{\star }=\frac{{\sum }_{\ell =1}^{N}{({p}_{\ell })}^{2}-{\sum }_{\ell =1}^{N}{P}_{\ell }^{\infty }{p}_{\ell }}{{\sum }_{\ell =1}^{N}{({p}_{\ell })}^{2}-\frac{1}{N}}\qquad {\rm{with}}\qquad {p}_{\ell }=\frac{{k}_{\ell }^{({\rm{in}})}}{{\mathcal{T}}}.$$

In Fig. 7(b) we illustrate the approximation given by Eq. 11, for *q* = 0 and q^*^ = 0.062, obtained for the best fit. However, Eq. 11 is a mean field result and important deviations may appear^10,44–46^. The result given by Eq. 11 makes sense in the description of taxis since the importance of a high demand zone can be defined in terms of the number of taxi trips *k*^(in)^ that arrive at this specific location. For example, in our schematic illustration presented in Fig. 1(a), now we understand that the bars with the value *k*^(in)^ determine the importance of the zones.

The transition probability matrix **W**^(out)^ defined in Eq. 6 captures all the information about the system’s global activity. We think that an OD rank of the zones defined as ${\overrightarrow{{\bf{P}}}}^{\infty }$ can be a valuable measure in the analysis of different transportation systems and a complement to other types of ranking algorithms introduced to determine location attractiveness incorporating geographic considerations into the PageRank algorithm^47–49^.

### Random walk strategy

**Figure 8:**
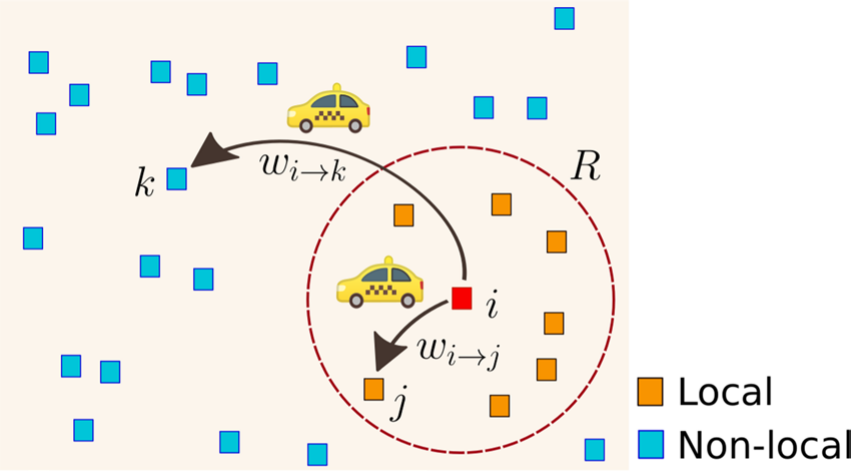
A schematic illustration of the mobility of taxis between high demand zones. There are two types of trips from a particular location *i*: First, to a site *j* inside a circular region of radius *R* centered in the location *i*, the probabilities to have a trip to these zones are constant; and, second, a trip to a zone *k* outside the circle of radius *R*. In this case, the probability to have this long-range movement decays as a power law with an exponential cutoff proportional to ${e}^{-\beta ({d}_{ik}-R)}{d}_{ik}^{-1}$, where *d*_*ik*_ is the geographical distance between *i* and *k*.

The results obtained before for the relationship of the transition probabilities describing the flow of taxis between zones and the geographical distances separating these locations, suggest that the spatial dynamics can be approximately described by a model with constant transitions to zones in a local neighborhood within a distance *R*, and a long-range dynamics defined by probabilities of transition proportional to ${e}^{-\beta ({d}_{ij}-R)}{d}_{ij}^{-1}$. The analysis of more than a billion trips reveals a particular emergent pattern in the spatial activity. The movement of taxis between high demand zones can be classified into two types of trips with particular characteristics illustrated in Fig. 8. We have local displacements for which a taxi departs from a high demand site and the probability of moving to another site of high activity is independent of the distance that separates them if they are located at a distance less than a value *R*. On the other hand, there may also be long-range displacements for which the separation between origin and destinations require distances greater than *R*. For this type of movements, we find that the probability of having a long-range trip depends on the distance and these particular transitions have characteristics observed in truncated Lévy flights.

In this way, to describe the global activity of the taxi’s mobility we use the model:

13 $${w}_{i\to j}^{({\rm{model}})}(R,\beta )=\frac{{\Omega }_{ij}(R,\beta )}{{\sum }_{\ell =1}^{N}{\Omega }_{i\ell }(R,\beta )},$$

where:

14 $${\Omega }_{ij}(R,\beta )=\left\{\begin{array}{ll}1 & {\rm{for}}\quad 0\ \le \ {d}_{ij}\ \le \ R,\\ \left(R/{d}_{ij}\right){e}^{-\beta ({d}_{ij}-R)} & {\rm{for}}\quad R < {d}_{ij}.\end{array}\right.$$

In this model, *β* and *R* are positive real parameters. The transition probabilities defined in Eqs. 13 and 14 are illustrated in Fig. 8. The radius *R* determines a neighborhood around each zone where the trips occur with equal probability to move from the initial site to any of the high demand zones in this region. Therefore, the displacements are independent of the geographical distance between origin and destination. That is, if there are *S* sites inside a circle of radius *R*, the probability of going to any of these sites is uniform. Additionally, for places beyond the local neighborhood, for distances greater than *R*, the transition probability decays as a power law with an exponential cutoff of the distance and is proportional to ${e}^{-\beta ({d}_{ij}-R)}{d}_{ij}^{-1}$. In this way, the parameter *R* defines a characteristic length of the local neighborhood and *β* controls the probability to have long-range displacements. In particular, in the limit *β* → *∞* the dynamics becomes local. We introduced a similar model with long-range transitions proportional to ${d}_{ij}^{-\alpha }$ (*α* > 0) in reference^8^ in the context of human mobility and encounter networks. In this case, the resulting dynamics can be similar to a rank model^50–52^ and a gravity model^3,53–55^. It is worth mentioning that the inverse of the parameter *β* in Eq. 14 gives us a characteristic distance; this exponential cutoff takes into account the finite size effect associated with a finite system like New York City.

In our previous analysis in Fig. 5, we found that *R* ≈ 1.8 Km. This value defines what we understand as a local neighborhood for this transport system. On the other hand, for distances *d*_*ij*_ > *R*, the probability to have a trip to a zone is highly influenced by the geographical distance and this long-range dynamics is determined by the values ${e}^{-\beta ({d}_{ij}-R)}{d}_{ij}^{-1}$ with *β* = 0.15 Km^−1^.

In the following part, we explore the predictions of this model for the annual global activity of taxi displacements in New York City by using the parameters *R* = 1.8 Km and *β* = 0.15 Km^−1^ found in the analysis of the taxi’s flow between high demand zones. In addition to Eqs. 13 and 14, that model the displacement between high demand zones, it is important to consider that these zones have different relevance in the whole dynamics, i.e., a trip can start from different zones with non-uniform probabilities. This fact is well described by the values of the out-degree ${k}_{i}^{({\rm{out}})}$ defined in Eq. 2 that gives the number of trips with origin in the zone *i*. In addition, from the results in Fig. 4, we know that the values ${k}_{i}^{({\rm{out}})}$ follow a hierarchical distribution with probabilities that decay as *p*(*k*) ∝ *k*^−*γ*^*e*^−*λk*^ where *k* represent the values of the out-degree. This result is observed in the annual datasets from 2009-2015. In this way, we simulate the dynamics of multiple taxis that start from an initial zone chosen randomly with a probability proportional to the values ${\{{k}_{i}^{({\rm{out}})}\}}_{i=1}^{N}$ that quantify the importance of each zone in the city. Then, a displacement is generated randomly from the origin site to a final zone by using the transition probabilities in Eq. 13; this algorithm is repeated to generate, through Monte Carlo simulations, the same number of displacements between high demand zones, as reported in Table 1.

**Figure 9:**
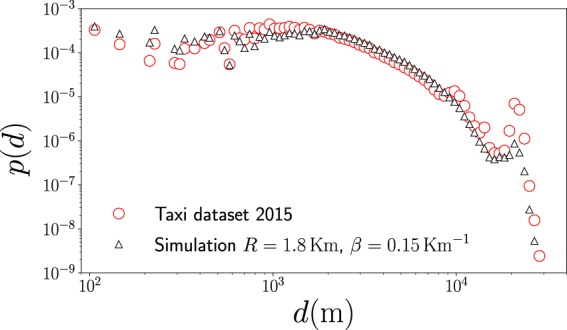
Statistical analysis of displacements of taxi trips in New York City. We depict the probability density *p*(*d*) of the geographical distance *d* between the departure zone and the final destination of taxis. We present statistics obtained from the analysis of the complete dataset for displacements in 2015 and data generated by using Monte Carlo simulations with transition probabilities ${w}_{i\to j}^{({\rm{model}})}(R,\beta )$ defined by the our model in Eqs. 13 and 14 with *R* = 1.8 Km and *β* = 0.15 Km^−1^. In both cases we use logarithmic spaced bin counts for distances between 10^2^ m ≤ *d* ≤ 4 × 10^4^ m.

In Fig. 9 we present the statistical analysis of the taxi’s displacements *d* generated randomly and the real values considering the activity in New York City in 2015. In our simulation, we generate 128 984 657 random displacements following the model in Eq. 13, with *β* = 0.15 Km^−1^ and *R* = 1.8 Km. Our findings show an agreement between the predictions of the model and the real dynamics. However, we observe that the predictions do not agree with the real data around *d* = 10 Km and *d* = 20 Km; this is a consequence of the singular dynamics induced by the two airports in New York City. An accurate modeling capturing the effects of these very attractive sites in a city requires modifications to the model explored. This fact is also visible in Fig. 5 where, for distances around 20 Km, we see different values of the transition probability that are not described by a model with long-range trips following ${w}_{i\to j}^{({\rm{out}})}\propto {e}^{-\beta ({d}_{ij}-R)}{d}_{ij}^{-1}$.

**Figure 10:**
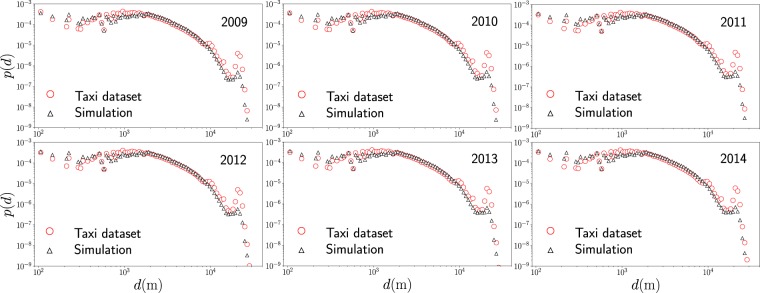
Probability density *p*(*d*) of the geographical distance *d* between the departure zone and final destination of taxis and the results generated through Monte Carlo simulations with transition probabilities between high demand zones ${w}_{i\to j}^{({\rm{model}})}(R,\beta )$ with *R* = 1.8 Km and *β* = 0.15 Km^−1^.

Finally, we repeat the same procedure to compare the predictions of the model with respect to the real data for taxi’s activity from 2009 to 2014. Our results for Monte Carlo simulations are presented in Fig. 10. We observe the same characteristics found in Fig. 9, with a good agreement between model and the data. The number of locations of high demand *N* and the number of displacements analyzed for each year are reported in detail in Table 1. The results in Table 1 also reveal that in average, in a year, approximately 43% of the trips are local movements for which the geographical distance *d* ≤ 1.8 Km, the rest of the trips are non-local with *d* > 1.8 Km.

## Discussion

In this research, we explore the massive records of more than one billion taxi-trips in New York City from January 2009 to December 2015. With this dataset of seven years, we generate an origin-destination matrix that has detail information of a vast number of trips. The mobility in New York City can be described as a directed weighted network that connects different zones of high demand for taxis. Each zone is characterized by the number of trips that arrive or depart from it and corresponds to nodes in the network. The arrivals and departures are the in-degrees and out-degrees of the directed network, and the flow gives different weights to the links of this spatial network.

We present a statistical analysis of the travel distance of each trip and found a long-range distribution that is almost the same for each of the seven years studied. On the other hand, the degree distributions, for the in and out degrees are, respectively, well modeled by a stretched exponential and a power law with an exponential cutoff. By defining the transition probabilities between zones, given by the origin-destination matrix and the out-degree, we are able to obtain a rank, called “*OD rank*”, analogous to the page rank of Google. We calculate the spectrum of eigenvalues and the main eigenvector, which is related to the in degree. The components of this eigenvector give the more relevant and attractive places in New York City, in terms of taxi trips.

The dependence of the transition probabilities with the distance between zones is obtained from the dataset, and based on that, we introduce a model that captures the global dynamics of trips. The data and the model describe, for short distances, a local dynamics independent of the spatial distance, and, for large distances, a dynamics that decays with distance as a power law with an exponential cutoff. The data agrees quantitatively with Monte Carlo simulations based on our model.

Finally, considering the taxi trips as a proxy of human mobility in cities, it might be possible that the long-range mobility and other features found for New York City would be rather general, and thus we expect a similar behavior in other large cities around the world for which these ideas can be applied as well.

## Methods

### Dataset description

In this section, we present a global description of the records explored to study the spatial dynamics in New York City. We use data for the activity of taxi trips from January 2009 to December 2015; these datasets are available to the public by the Taxi and Limousine Commission in the New York City open data website^56^. The data available include information for all taxi trips in New York City when the taxis are in service. The records comprise several fields capturing pick-up and drop-off dates and times, pick-up (origin) and drop-off (destination) locations, itemized fares, rate types, payment types and driver-reported passenger counts^56^.

**Figure 11:**
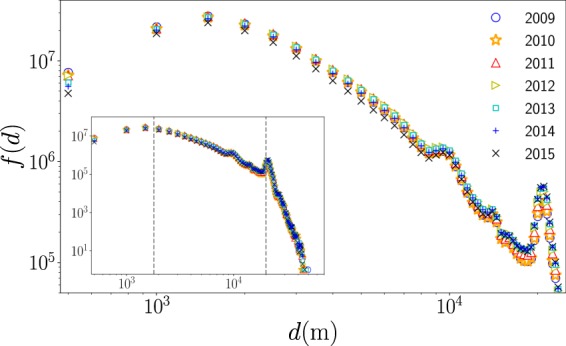
Statistics of displacements *d* of taxi trips in New York City. We depict the frequency *f*(*d*) of the geographical distance *d* between the origin and destination of taxis. The results are obtained from annual datasets between January 2009 to December 2015. In the inset, we present *f*(*d*) as a function of *d* for the analysis of all the distances with a scale in the frequencies that ranges from 10^0^ to 10^8^. The two vertical dashed lines represent *d* = 1.8 Km and *d* = 20 Km. Additional information about the datasets explored is presented in Table 2.

Now, to complement the information in Fig. 2, and identify other global characteristics in the taxi’s spatial activity, we analyze the geographical distance *d* between the origin and destinations in each trip calculated from the longitude and latitude coordinates of these locations reported in the database. Here, it is worth mentioning that other types of distances can be implemented; in particular, the distance of the path in the street network connecting origin and destinations. In fact, powerful techniques have been introduced exploring taxi trips in New York City to estimate the driving distance based on the origin and destination coordinates^59^. However, due to the different paths that a taxi can follow to carry out each trip, in the following we use the geographical distance *d*. In Fig. 11, we present the statistical analysis of the geographical distances *d*. We depict the frequencies *f*(*d*) of the displacements obtained from uniform bin counts with Δ*d* = 500 m for taxi trips. Different markers show the results for the analysis in a year. We can see that the frequencies *f*(*d*) maintain the same characteristics from 2009 to 2015, and the statistics reveal three important intervals: the first for *d* < 1.8 Km with higher values of the frequencies, a second interval for 1.8 Km ≤ *d* < 20 Km where *f*(*d*) gradually decays and finally, for distances around 20 Km, we identify a peak that decays rapidly with the distance; this peak is associated with large displacements from Manhattan to the JFK airport (as a reference, the geographical distance between Times Square and the JFK airport is 20.6 Km). In a similar way, we identify another relative maximum at *d* = 10 Km: this increase in the frequencies is associated with trips between Manhattan and La Guardia airport (with *d* = 9.8 Km between Times Square and this airport). These are examples of how important locations can induce long-range dynamics in the taxi’s mobility. In this case, the two airports in New York City influence the taxi transportation mode in the whole city. This important feature has been observed in other cities with airports located at the city’s periphery (a particular case is reported in^31^).

In Table 2, we summarize the global information found for the spatial dynamics per year. We present the number of taxi trips analyzed, the average distance, the largest distance traveled as well as the fraction of trips with distances at different intervals. From the information in this table, when we examine the complete records from 2009 to 2015, we observe that 41.33% of the taxi trips have displacements with *d* less than 1.8 Km, whereas a 57.49% of the trips involve long-range displacements in the interval 1.8 Km ≤ *d* < 20 Km, and only a 1.18% of the trips have *d* greater than 20 Km. The average displacement of trips is $\left\langle d\right\rangle =3.3\ {\rm{Km}}$ and the maximum value observed in the records is 51.87 Km. All these quantities give us a first characterization of the spatial activity of the taxi transportation mode.

**Table 2:** Taxi records and displacements in New York City. We analyze taxi trips records from January 2009 to December 2015. Here, ${\mathscr{T}}$ is the total number of trips, the length $\left\langle d\right\rangle$ is the average distance between the initial and final location whereas ${d}_{\max }$ is the length of the maximum displacement observed in each dataset. On the other hand, in the last three columns we include the fraction of displacements (as percentages) in the intervals 0 ≤ *d* < 1.8 Km, 1.8 Km ≤ *d* < 20 Km and for values of *d* larger than 20 Km. In the last row, we present the results obtained for the whole dataset.

Year	${\mathscr{T}}$	$\left\langle {\bf{d}}\right\rangle {\boldsymbol{(}}{\bf{Km}}{\boldsymbol{)}}$	${{\bf{d}}}_{{\bf{\max }}}{\boldsymbol{(}}{\bf{Km}}{\boldsymbol{)}}$	% 0 ≤ *d* < 1.8 Km	% 1.8 Km ≤ *d* < 20 Km	% *d* ≥ 20 Km
2009	167 165 746	3.14	49.02	42.76	56.32	0.92
2010	163 913 012	3.19	51.87	42.05	56.96	0.99
2011	171 166 041	3.27	45.16	41.05	57.85	1.1
2012	173 087 239	3.34	44.91	40.37	58.47	1.16
2013	168 937 296	3.36	47.67	40.45	58.28	1.27
2014	160 822 602	3.38	43.78	40.92	57.72	1.36
2015	142 958 901	3.41	45.95	41.82	56.62	1.56
2009–2015	1 148 050 837	3.30	51.87	41.33	57.49	1.18

### Geographical and shortest path distances

**Figure 12:**
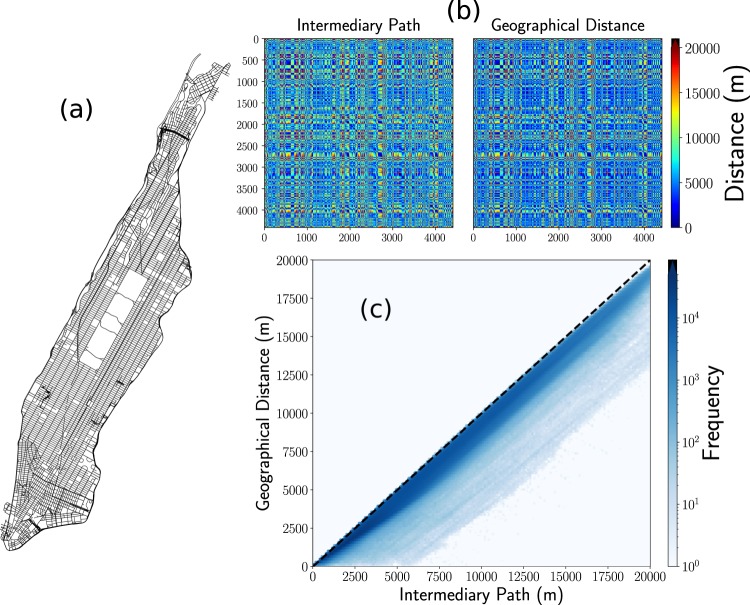
Distances between intersections in Manhattan. (**a**) Manhattan’s street network, (**b**) Distance matrices for 4 409 intersections in this network. We depict the results for the length of the intermediary path and the geographical distance between these intersections; the distances are indicated by the colorbar. In (**c**) we present the hexagonal bin counts for the geographical distances and the respective length of the intermediary path. We depict a dashed line, with unit slope, that represents the case when the two distances are the same. Clearly, since the intermediary path is always greater or equal than the geographic distance, we only have data in the lower triangle of the figure. We show with a colorbar the frequencies for the values found in each bin. The street map, intersections, and intermediary paths were obtained and analyzed with the OSMnx package^57,58^.

In the analysis of the information described before, we use the geographical distance *d* between origin and destination. This election is based on the fact that we only know the geographical coordinates of origins and destinations for each trip. However, another important quantity to consider is the length of the intermediary path that the vehicle follows on the street network. The information of the street network, the length, and direction of each street and all the intersections, can be obtained from different sources like OpenStreetMap^60^ or generated by using specialized algorithms (see for example^59^). In general, the length of the geographical distance is less or equal than the length of the shortest path between two points in a city. In Fig. 12 we explore this relation for all the intersections in Manhattan’s street network. We analyze the information available in OpenStreetMap^60^ and the OSMnx Python package^57,58^ to generate the street network depicted in Fig. 12(a). From this structure, we obtain the geographical coordinates of 4 409 intersections. In Fig. 12(b) we calculate all the distances between these intersections, taking into account the length of the intermediary path, and the respective geographical distance. The results are presented as matrices for which the entry *l*, *m* represents the respective distance between intersections *l* and *m*. The two matrices are similar; however, the matrix with intermediary paths is asymmetric since it includes the directions of the streets. In Fig. 12(c) we explore the relation between the two distances by plotting all the values presented in Fig. 12(b). The results reveal that a high fraction of the values is close to a linear relation. Similar results apply for the whole city and, in this way, the main features of the global activity of taxis can be analyzed by using only geographical distances between origin and destination. However, in other contexts, a description of the complete path followed by the vehicle is necessary. See refs. ^35,59^ for a detailed discussion and models for taxi’s mobility at the level of intermediary paths.
